# Toward an improved conceptualization of emotions in patients with cancer

**DOI:** 10.3389/fpsyt.2024.1352026

**Published:** 2024-03-27

**Authors:** Joost Dekker, Elise Doppenberg-Smit, Annemarie Braamse, Femke Lamers, Myra van Linde, Henk M. W. Verheul, Mirjam Sprangers, Aartjan T. F. Beekman

**Affiliations:** ^1^ Department of Psychiatry, Amsterdam University Medical Center, Vrije Universiteit Amsterdam, Amsterdam, Netherlands; ^2^ Amsterdam Public Health, Mental Health Program, Amsterdam, Netherlands; ^3^ Cancer Centre Amsterdam, Cancer Treatment and Quality of Life, Amsterdam, Netherlands; ^4^ Department of Medical Psychology, Amsterdam University Medical Center, University of Amsterdam, Amsterdam, Netherlands; ^5^ Department of Medical Oncology, Amsterdam University Medical Center, Vrije Universiteit Amsterdam, Amsterdam, Netherlands; ^6^ Department of Medical Oncology, Erasmus University Rotterdam, Erasmus Medical Center Cancer Institute, Rotterdam, Netherlands

**Keywords:** cancer, distress, emotion, mental disorder, mental health, theory

## Abstract

Cancer and its associated treatment is a major stressor, leading to emotions such as anxiety or depressive mood. Human emotions have developed through the course of evolution because they facilitate adaptation to important events, such as cancer and its associated treatment. On the other hand, emotions can be maladaptive and interfere with adaptation to cancer. Emotions are maladaptive if they are disproportionally severe or persistent, and if they interfere with functioning. We aim to expand the conceptualization of adaptive and maladaptive emotions in patients with cancer. We draw on major theories in the field of mental disorder and mental health, and apply these theories to conceptualize adaptive and maladaptive emotions in patients with cancer. (i) Maladaptive emotions have two essential features: mental dysfunction and patient harm. Maladaptive emotions are characterized by a network of strongly associated emotional symptoms, which may include cancer-related somatic symptoms. The dysfunctional symptom network is hypothesized to be the result of disturbance of life goal pursuit caused by cancer. (ii) Adaptive emotions have two essential features: ability to deal with cancer and functioning well. The ability to use emotions in an adaptive way depends on skills to recognize, express, and regulate emotions in a flexible manner. A secure attachment style facilitates adaptive emotional responses to cancer. The present conceptualization of adaptive and maladaptive emotions is expected to contribute to better understanding and management of emotions in patients with cancer.

## Introduction

Cancer and its associated treatment is generally accompanied by emotions such as fear, anxiety, sadness, low mood or anger (1). In the field of psycho-oncology, emotions are frequently conceptualized in terms of distress. The concept of ‘distress’ refers to ‘a multifactorial unpleasant emotional experience of a psychological (i.e., cognitive, behavioral, emotional), social and/or spiritual nature that may interfere with the ability to cope effectively with cancer, its physical symptoms, or its treatment’ (1). The concept of cancer-related distress has fueled development of the field of psychosocial cancer care (2). However, increasing evidence suggests that the conceptualization of emotions in cancer needs to be improved, as current conceptualization is suboptimal.

A key issue is that distress does not adequately differentiate between patients who need mental health care and those who do not. A range of studies in various countries have shown that the majority of patients scoring above the validated cut off for distress do not accept professional mental health care (3–6). For example, in a multicenter study patients with metastatic colorectal cancer were screened at three time points, a trained nurse discussed the screening outcome and offered a stepped mental health care program (consisting of guided self-help, face-to-face problem-solving therapy, or a referral to professional mental health care). While 60% of patients showed elevated distress during at least one of the screening time points, a mere 11% of patients accepted mental health care (6). ‘No need for psychosocial services and support’ is the most frequently reported reason for not accepting mental health care (7). Qualitative studies further explored patients’ attitudes and expectations about distress management. Patients indicated a preference to deal with distress themselves and with support from relatives and friends (8), the clinical team (9), or other patients with cancer (10). Other explanations for patients not accepting mental health care include stigma linked to mental health issues, transportation problems, and lack of confidence in services (7). Regardless, the fact that ‘no need’ is the most frequently cited reason for not seeking professional mental health care and that many patients report they wish to manage distress outside of professional mental health care suggest that a fundamental reconceptualization of distress and distress management is warranted (11).

We have argued that distress as such (that is, a high intensity of emotions such as sadness, fear, anxiety or anger) is not a sufficient reason for professional mental health care. Instead, a distinction between adaptive and maladaptive emotions is needed (11–13). Human emotions have developed through the course of evolution because they facilitate adaptation to important events (14). Emotions alert, motivate and prepare us to deal with these events (15). For example, fear causes cognitive, behavioral and physiological changes which help to face a threatening event (14). Sadness turns our attention inwards, promoting resignation and acceptance. The expression of sadness may elicit sympathy and support from other people (16). Emotions are essentially *adaptive* – they help us to adapt to events in the environment (13). On the other hand, emotions sometimes hamper adaptation, leading to significant distress and disability (17). Emotions are *maladaptive* if they are disproportionally severe or persistent, and if they interfere with functioning. Examples include severe anxiety leading to the avoidance of medical treatment, or depression leading to lack of motivation to continue treatment.

Likewise, emotions are a natural reaction to the threat posed by a cancer diagnosis and the burden of cancer treatment. These emotions may help patients cope with cancer and are thus adaptive. Presumably, patients with adaptive emotions prefer to deal with emotions outside of professional mental health care - on their own, and with emotional support from relatives, friends, peers, and care-givers (i.e., doctors and nurses) (12). In some patients emotions develop in a maladaptive manner. Emotions are maladaptive if they are disproportionally severe or persistent, and if they interfere with functioning. We have argued that patients with maladaptive emotions need professional mental health care (12). Hence, distinguishing between adaptive and maladaptive emotions is key to identifying patients who are in need of professional mental health care. It should be acknowledged that the definition of distress states that distress ‘may interfere with the ability to cope effectively with cancer, its physical symptoms, or its treatment’ (1). However, this feature is not used to distinguish between patients who do or do not need professional mental health care, whereas we consider this point to be of crucial importance (see Bai (18) for a similar point of view).

To distinguish between adaptive and maladaptive emotions, a standardized psychiatric interview such as the Composite International Diagnostic Interview (CIDI) can be used (19). However, the CIDI categorizes mental disorders, without a special focus on emotions. Alternatively, one can ask patients whether they need professional mental health care related to their emotional problems. To standardize this, the Problem List can be used (20–22). Although the patient experience is important, a more scientifically informed distinction between adaptive and maladaptive emotions is desirable. We argue that the conceptualization of adaptive and maladaptive emotions needs to be improved in order to derive indicators for distinguishing between them.

So far, we have characterized adaptive and maladaptive emotions as emotions that either support or interfere with the process of adaptation to cancer, respectively. In the present paper, we aim to further expand this conceptualization of adaptive and maladaptive emotions in patients with cancer. Specifically, (i) we characterize the nature of adaptive and maladaptive emotions; and (ii) we identify processes that cause emotions to develop in either an adaptive or maladaptive direction, focusing on patients with cancer. To provide the background information needed to put this conceptualization into perspective, we first briefly review the process of adaptation to disease, as well as the role of emotions in this process. Next, we draw on major theories in the field of mental disorder and mental health, and apply these theories to conceptualize adaptive and maladaptive emotions in patients with cancer. Although in reality the distinction may be gradual, for ease of discussion we make a dichotomous distinction between adaptive versus maladaptive emotions. This is in line with clinical practice, in which a dichotomous decision must be made about whether or not to refer to professional mental health care.

## Adaptation to disease

**Figure 1:**
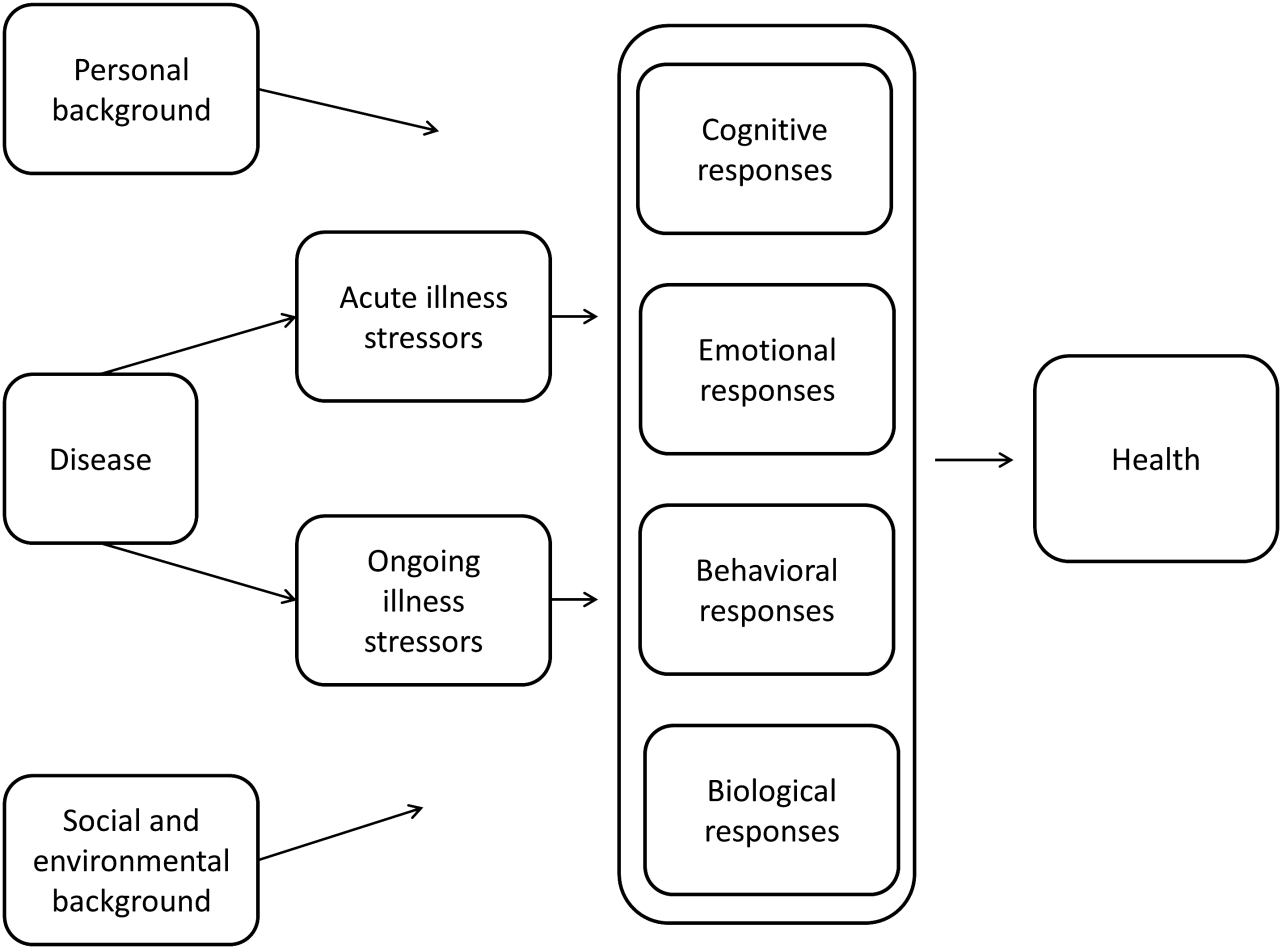
Model of psychological adaptation to chronic disease (27, 28).

Disease and its associated treatment constitute a source of stress for many patients. The experience of disease-related stress elicits a process of adaptation (or adjustment). This process has been conceptualized in several ways, including the stress-coping model (23), the illness representation model (24), the adaptive tasks and coping model (25), and the adjustment model (26). We have previously provided an integrated summary of these models (27, 28), as presented in **Figure 1**. Briefly, disease induces both acute illness stressors (e.g., learning the disease diagnosis, undergoing burdensome treatment, or experiencing a relapse) and ongoing illness stressors (e.g., pain, fatigue, or poor physical functioning). Acute and ongoing illness stressors induce loosely coupled cognitive, emotional, behavioral, and biological responses that influence health outcomes. Examples include catastrophizing thoughts (cognitive response), depressive symptoms (emotional response), avoidance of activity (behavioral response), and (chronic) activation of the hypothalamic-pituitary-adrenal axis (biological response). Background factors moderate cognitive, emotional, behavioral, and biological responses to these acute and ongoing stressors. These background factors include the patient’s personal background (e.g. personality, life goals) as well as the social and environmental background (e.g. support from family and friends, socio-economic status, neighborhood).

The emotional response to stressors is essentially adaptive. Emotions have developed over the course of evolution because they facilitate adaptation to important events in the environment (14). Emotions alert, motivate, and prepare humans to deal with such events (15). Cancer and its related treatment constitute such a salient event: emotions in response to cancer may help patients to cope with cancer (11, 12).

However, emotions may interfere with the adaptation process and are thus maladaptive. Mood and anxiety disorders have a major, negative impact on cognitive function, psychological well-being, activities of daily living, or social and occupational functioning (29). These negative effects of mood and anxiety disorders also occur in patients with cancer (1). In addition, emotions may interfere with cancer treatment: mood and anxiety disorders can impede medical communication, decision making, and adherence (1).

The model in **Figure 1** emphasizes that emotions cannot be conceptualized in isolation. On the contrary, emotions interact with a range of other factors. The present paper focuses on emotions in interaction with factors in the patient’s personal background. The interaction of emotions with cognitive, behavioral and biological responses, as well as with personal and social background, has been reviewed elsewhere (30–33). We start with the conceptualization of maladaptive emotions and processes that cause emotions to develop in a maladaptive direction, as most theoretical work has been done in this area. We then turn to the conceptualization of adaptive emotions and causal factors.

## Maladaptive emotions

To conceptualize maladaptive emotions of patients with cancer, the theoretical framework of the Diagnostic and Statistical Manual of Mental Disorders, Fifth edition can be used (DSM-5 (17)). The DSM-5 provides a general definition of mental disorder along with detailed criteria for specific disorders. Depressive disorders, anxiety disorders, and trauma- and stressor-related disorders, as well as adjustment disorder (included in a previous version of the DSM (34)) are common in patients with cancer (35, 36).

According to DSM-5, the general definition of a mental disorder has two essential features. *First*, ‘a mental disorder is a syndrome characterized by clinically significant disturbance in an individual’s cognition, emotion regulation, or behavior that reflects a dysfunction in the psychological, biological, or development processes underlying mental functioning’ (17). In earlier work, mental dysfunction has been described as ‘the inability of some mental mechanism to perform its natural function’ (37) or ‘organismic dysfunction’ (38, 39). The nature of this dysfunction is far from being clarified. We will return to this issue in the next section. For now, this first feature suggests that maladaptive emotions in response to cancer reflect a dysfunction in mental functioning. *Second*, ‘mental disorders are usually associated with significant distress or disability in social, occupational, or other important activities’ (17). In earlier work, concepts such as ‘distress, disability, or certain other types of disadvantage’ (38, 39) and ‘harm’ (37, 40) have been used to refer to this feature of mental disorders. With regard to maladaptive emotions, this second feature implies that maladaptive emotions are associated with harm (that is, significant distress or disability in social, occupational, or other important activities).

In foundational work, Wakefield combined the two features of a mental disorder into a ‘harmful mental dysfunction’, that is, a mental dysfunction that causes significant harm to the person (37). Wakefield argued that both ‘mental dysfunction’ and ‘harm’ are required for a condition to qualify as a mental disorder (37), although in specific cases this requirement may need careful consideration (40). This leads us to characterize *maladaptive emotions in patients with cancer* as a disturbance in a patient’s emotional response to cancer that reflects a dysfunction in mental functioning and causes harm to the patient.

### Dysfunction

*Network approach.* The nature and causes of mental dysfunction have been conceptualized in numerous ways (41). Here we focus on the relatively new network approach, which is well suited to study mental disorders in patients with a somatic disease (42) (see next section), and is compatible with traditional approaches (43).

According to the network approach (44), an external event (e.g., an adverse life event) may activate symptoms (e.g., depressed mood, feeling agitated, or feeling anxious). Symptoms are thought to form a network: symptoms interact and activate each other. Graphically, symptoms are described as nodes and causal interactions between symptoms are the connections between nodes. A central tenet of the network approach is that strong connections among symptoms (i.e., a strong network) cause symptoms to activate each other and to remain activated, even after the external stressor has been controlled. This results in a syndromic state, which is resilient to change. This state constitutes a mental disorder, e.g., depressive disorder. Conversely, if strong connections among symptoms are not present, symptoms will gradually subside. Evidence supporting this theory is accumulating (45).

Applying the central tenet of the network approach to emotions in patients with cancer leads to our first hypothesis: maladaptive emotions in patients with cancer are characterized by stronger associations between emotional symptoms, compared to adaptive emotions.

*Somatic symptoms.* For many diagnoses, the DSM-5 requires that symptoms are not attributable to the physiological effects of a substance (e.g., drugs, medication) or another medical condition (17). For patients with a medical condition such as cancer and its treatment, this requirement is not realistic: symptoms may indeed be associated with both the underlying medical condition and the mental disorder. A prime example is fatigue, which is associated with cancer(treatment) as well as depression. Other examples include weight loss and concentration problems.

It has been argued that the network approach provides a better paradigm to study mental disorders in patients with medical conditions such as cancer than traditional DSM-5 criteria (42). Symptoms associated with both the medical condition and the mental disorder may act as ‘bridge symptoms’: the shared symptom is activated by the medical condition, which in turn activates the network of mental disorder, and vice versa. For example, fatigue due to cancer(treatment) activates other symptoms of depression, such as depressed mood, or feelings of worthlessness.

Above, we hypothesized that maladaptive emotions in patients with cancer are characterized by stronger associations among emotional symptoms. Extending this premise to include somatic, cancer(treatment)-related symptoms leads us to hypothesize that somatic symptoms contribute to emotions being maladaptive, if the somatic symptoms are included in a strong network of emotional symptoms. Stated more formally, our second hypothesis is: maladaptive emotions in patients with cancer are characterized by stronger associations between cancer(treatment)-related symptoms and emotional symptoms, compared to adaptive emotions. There is some evidence to support this hypothesis (46).

*Goal disturbance.* Above, we have characterized the nature of maladaptive emotions in patients with cancer. The next crucial question is to determine which processes cause emotions to develop in a maladaptive direction.

Although frequently presented as an alternative, the network approach is actually compatible with traditional approaches toward mental disorder. The onset of a mental disorder can be governed by a process assumed in traditional theory, while its maintenance is fueled by direct interactions between symptoms (43). Based on human self-regulation theory (47), Strauman developed this type of hybrid model of the onset of depression (48, 49). Strauman built on the well-validated idea of two distinct brain/behavior systems for goal pursuit: the promotion system that operates to maximize positive outcomes, and the prevention system that operates to minimize negative outcomes. Strauman hypothesizes that frequent failure to achieve goals leads to hypo-activation of the promotion system, and thereby to depressive symptoms. Additionally, hypo-activation of the promotion system leads to hyper-activation of the prevention system, resulting in symptoms of anxiety and agitation. Once a network of depressive, anxious, and agitated symptoms has been formed, it remains activated through interactions among the network’s symptoms. In this hybrid model, failure to achieve goals leads to a strong network of depressive, anxious, and agitated symptoms; the network is maintained through reciprocal activation among symptoms.

Patients with cancer frequently experience disturbance of life goal pursuit (50). Disturbances may occur in the pursuit of work/study-related goals, health-related goals, social goals, psychological goals, or leisure/pleasure-related goals (51). Strauman’s theory suggests that frequent failure to achieve these life goals leads to a strong network of depressive or anxious symptoms. For example, frequent failure to achieve work-related goals is hypothesized to result in a strong network of depressive symptoms and the maladaptive emotions of a depressive disorder. This leads to our third hypothesis: in patients with cancer, disturbance of life goal pursuit is associated with a stronger network of emotional symptoms.

Individuals differ in their emotional response to cancer (52). A history of mental health problems has been consistently found to be associated with an increased risk of distress (52). Within the network/self-regulation theory this finding can be interpreted as follows: patients with prior mental disorders are vulnerable, in the sense that a network of strongly associated emotional symptoms can be easily re-established once these associations have been primed. Cancer(treatment) may induce goal disturbance which re-establishes a previously formed network of emotional symptoms in these patients. This leads to our fourth hypothesis, that a history of prior mental disorders moderates the association between goal disturbance and maladaptive emotions: the association between disturbance of life goal pursuit and a stronger network of emotional symptoms is more pronounced in patients with cancer and prior mental disorders, compared to patients without prior mental disorders.

### Harm

We now turn to discussing harm, the second essential feature of maladaptive emotions. DSM-5 defines harm as ‘significant distress or disability in social, occupational, or other important activities’ (17). Although this is a broad definition, DSM-5 does not provide a conceptualization of the dimensions of harm, and neither do other authors (37, 39, 40). This may lead to confusion regarding which dimensions to consider when evaluating harm.

**Figure 2:**
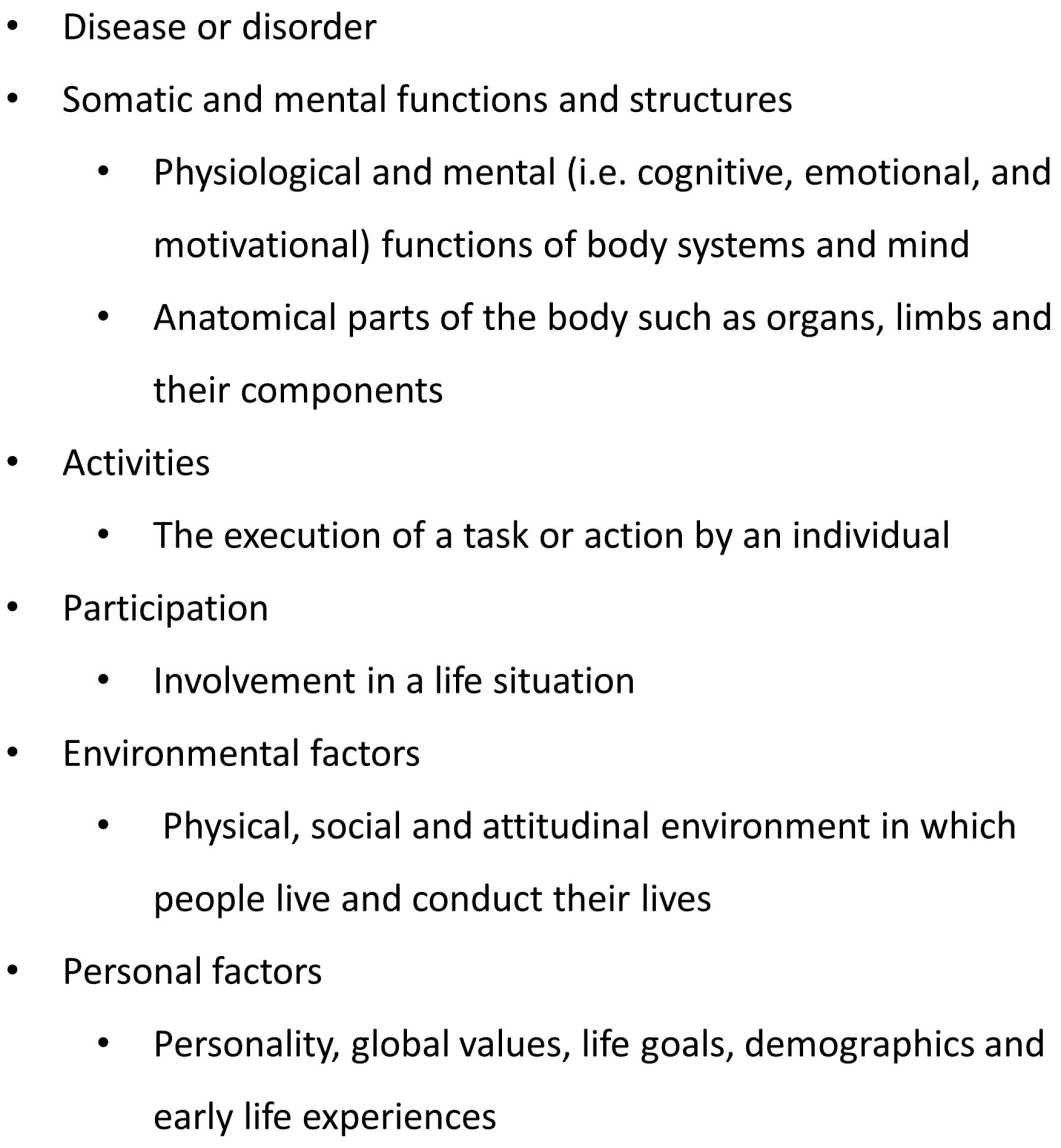
Health domains (54).

The World Health Organization has conceptualized domains of functioning in the International Classification of Functioning (ICF) (53). Drawing on this classification and a proposal to more explicitly articulate psychological components of this classification (27), a number of health domains can be distinguished (54), as listed in **Figure 2**. The ICF describes these dimensions in greater detail (53). We suggest that this classification can be used to more systematically evaluate harm associated with maladaptive emotions. This leads to our fifth hypothesis: maladaptive emotions in patients with cancer are associated with problems in somatic and mental (i.e. cognitive, emotional, and motivational) functions, activities, participation, social and environmental factors, or personal factors, compared to adaptive emotions.

## Adaptive emotions

Theoretical work to define mental health is of a fairly recent date. Reviewing the literature, Manwell et al. (55) reported the results of a mixed methods multidisciplinary international survey on the definition of mental health. They proposed the following as the core concept of mental health: ‘the ability or capacity of a person to effectively deal with his/her environment, resulting in the subjective experience of understanding and managing stressors’. Their definition corresponds well with that of Galderisi et al. (56), who consider mental health to ‘encompass a range of skills, resulting in a dynamic state of internal equilibrium which enables individuals to use their abilities in harmony with universal values of society’. Both definitions refer to two essential features of mental health, which we shall refer to as ‘ability’ (or skill) and ‘functioning well’. This leads us to characterize *adaptive emotions in patients with cancer* as the emotional component of a patient’s ability to deal with cancer which enables the patient to function well.

### Ability

Emotions can contribute to the ability to deal effectively with cancer if the patient possesses a variety of skills. According to Galderisi et al. (56), an individual needs to be able to recognize, express, and regulate emotions, and importantly, to regulate emotions in a flexible manner. The concept of ‘emotion regulation flexibility’ derives from psychological flexibility theory (57, 58). Successful adaptation depends on the ability to flexibly adjust emotions in accordance with situational demands, supporting the pursuit of personally meaningful goals (59, 60). Importantly, flexibility theory ‘does not assume that reducing distress is the desired outcome of a regulatory response. Reducing distress is only functional to the extent that doing so facilitates the pursuit of self‐endorsed, meaningful, valued goals’ (58) (page 2). Being open and accepting of emotional experiences, even emotional experiences with a negative valence such as sadness or fear, may thus facilitate the pursuit of goals (57, 61). For example, cancer-related emotions such as fear or sadness carry the potential to facilitate effective adaptation by increasing adherence to medical regimens or by eliciting social support. Applying these notions to emotions in patients with cancer leads to our sixth hypothesis: patients with cancer with adaptive emotions are characterized by a flexible regulation of emotions, compared to patients with maladaptive emotions.

*Attachment.* As with maladaptive emotions, a crucial question is which processes cause emotions to develop in an adaptive direction. Few theories have focused on factors that causally contribute to the development of adaptive emotions. Of the theories explaining adaptive emotions, the attachment theory is most developed and investigated, yielding useful results (62). In early childhood, through interactions with the primary caregiver, the individual may develop a secure attachment style characterized by confidence that others will be available in times of stress and the ability to self-regulate stressors (63). A secure attachment style has been found to be associated with better adaptation, growth, well-being, and resilience, both in patients with chronic illness in general as well as in patients with cancer (63–67). We hypothesize that a secure attachment style also enables patients to have adaptive emotional responses to cancer. This leads to our seventh hypothesis: patients with cancer with adaptive emotions are characterized by a secure attachment style, compared to patients with maladaptive emotions.

### Functioning well

Above, we used the ICF to define health domains in which mental disorder may cause harm. The same domains can be used to evaluate functioning well (see **Figure 2**). This leads to our eighth hypothesis: adaptive emotions in patients with cancer are associated with positive outcomes on somatic and mental (i.e. cognitive, emotional, and motivational) functions, activities, participation, social and environmental factors, or personal factors, compared to maladaptive emotions.

## Discussion

**Figure 3:**
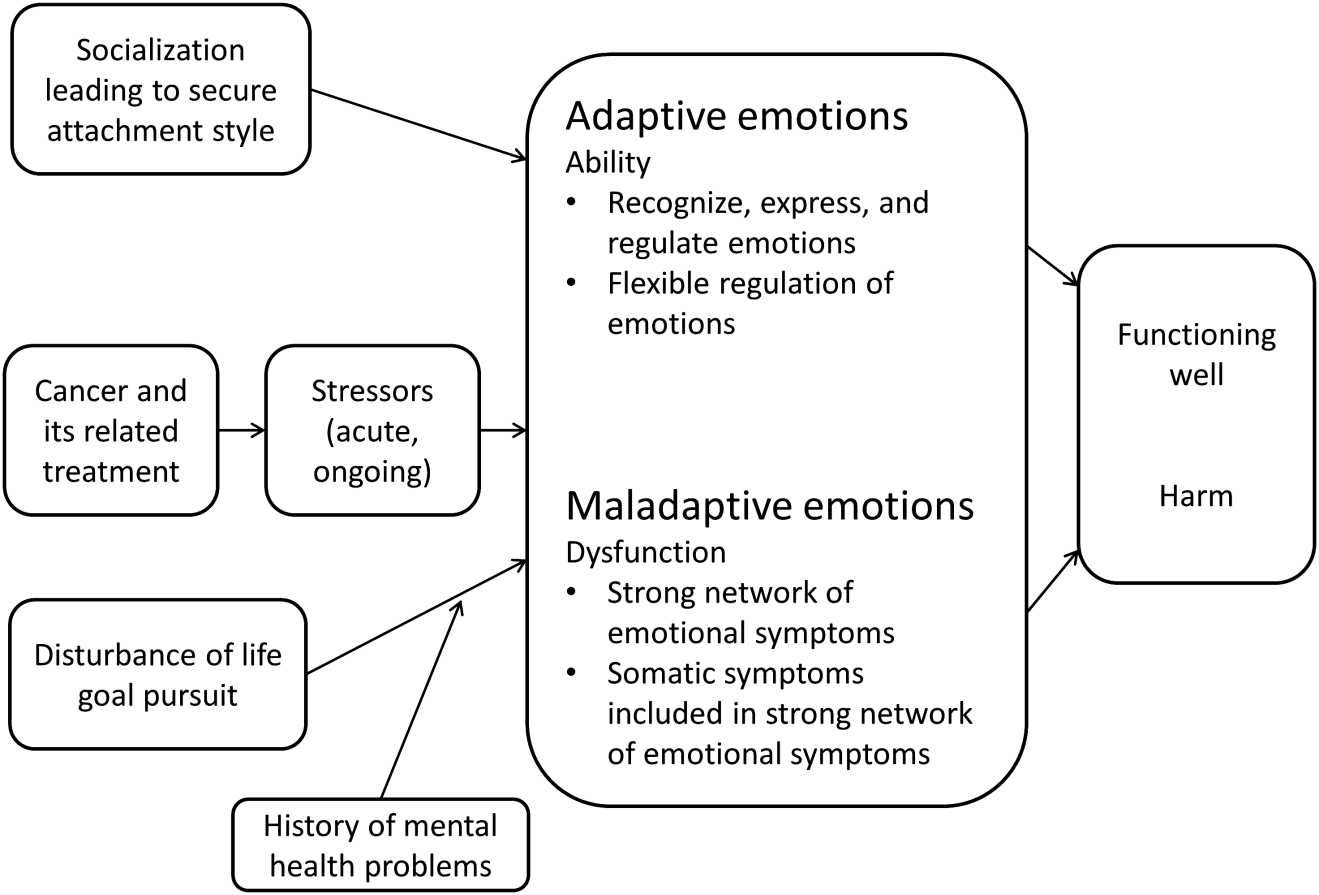
Conceptualization of adaptive and maladaptive emotions in patients with cancer.

In summary, cancer and its associated treatment is a major stressor, inducing emotional and other responses. Cancer-related emotions may be either adaptive or maladaptive; that is, they support or interfere with the process of adaptation to cancer, respectively. The above conceptual analysis of adaptive or maladaptive emotions in patients with cancer leads to the following conclusions. (i) Maladaptive emotions are a disturbance in a patient’s emotional response to cancer that reflects a dysfunction in mental functioning and causes harm to the patient. The dysfunction in the processes underlying emotional functioning can be conceptualized in terms of the network approach toward mental disorders: maladaptive emotions are characterized by strong associations among emotional symptoms and between emotional and cancer(treatment)-related somatic symptoms. The dysfunctional network of emotional symptoms is hypothesized to be the result of disturbance of life goal pursuit (e.g., work/study related goals) caused by cancer and its associated treatment. A history of prior mental disorders increases the risk for maladaptive emotions, because goal disturbance can reactivate a previously formed dysfunctional network of emotional symptoms. Maladaptive emotions in patients with cancer are associated with problems in various health domains. (ii) Adaptive emotions are the emotional component of a patient’s ability to deal with cancer that enables the patient to function well. The ability to use emotions in an adaptive way depends on skills to recognize, express, and regulate emotions, as well as to regulate emotions in a flexible manner. A secure attachment style promotes patient’s ability to have adaptive emotional responses to cancer. This conceptualization of adaptive and maladaptive emotions in patients with cancer is summarized in **Figure 3**. We have derived eight specific hypotheses, some of which are currently being tested. **Table 1** provides an overview of the hypotheses on maladaptive and adaptive emotions, respectively, arranged according to the nature of and causal processes affecting these emotions.

**Table 1:** Hypotheses on adaptive and maladaptive emotions in patients with cancer.

Adaptive emotions
Nature
• Patients with cancer with adaptive emotions are characterized by a flexible regulation of emotions, compared to patients with maladaptive emotions.
• Adaptive emotions in patients with cancer are associated with positive outcomes on somatic and mental (i.e. cognitive, emotional, and motivational) functions; activities; participation; social and environmental factors; or personal factors, compared to maladaptive emotions.
Causal processes
• Patients with cancer with adaptive emotions are characterized by a secure attachment style, compared to patients with maladaptive emotions.
Maladaptive emotions
Nature
• Maladaptive emotions in patients with cancer are characterized by stronger associations between emotional symptoms, compared to adaptive emotions.
• Maladaptive emotions in patients with cancer are characterized by stronger associations between cancer(treatment)-related symptoms and emotional symptoms, compared to adaptive emotions.
• Maladaptive emotions in patients with cancer are associated with problems in somatic and mental (i.e. cognitive, emotional, and motivational) functions, activities, participation, social and environmental factors, or personal factors, compared to adaptive emotions.
Causal processes
• In patients with cancer, disturbance of life goal pursuit is associated with a stronger network of emotional symptoms.
• The association between disturbance of life goal pursuit and a stronger network of emotional symptoms is more pronounced in patients with cancer and prior mental disorders, compared to patients without prior mental disorders.

This conceptualization pertains primarily to emotions with a negative valence, such as feeling anxious, down, or agitated. The definition of a mental disorder, the network approach, the theory of goal disturbance and the concept of emotion regulation flexibility all refer primarily to emotions with a negative valence. However, emotions with a positive valence such as feeling good, calm, and concentrated may also play an important role, for example in functioning well. The role of emotions with a positive valence needs further study.

While our conceptualization of adaptive and maladaptive emotions applies to patients with cancer, the main propositions in this framework may apply to patients with other diseases, such as cardiac disease or diabetes. We deliberately confined the present analysis to patients with cancer because other diseases may require other aspects to be included in the analysis; in particular, emotions and diseases such as cardiac disease and diabetes may share a common physiological basis (68, 69). Regarding patients with cancer, a common physiological basis for emotions and cancer seems less likely (70, 71). This allowed us to develop the conceptualization of adaptive and maladaptive emotions without the need to concern ourselves with the possibility of a common physiological basis. We encourage researchers to further expand our theoretical framework to conceptualize emotions associated with other diseases.

### Clinical implications

If adequately supported by empirical research, the present conceptualization can be used in clinical practice. First and foremost, indicators can be derived to distinguish between patients who do or do not need professional mental health care. For example, using a network approach the strength of (the network of) emotional and somatic symptoms could act as an indicator of the need for mental health care, rather than solely relying on intensity of distress. Patients with a strong network of emotional and somatic symptoms will likely experience maladaptive emotions and need professional mental health care, while others may primarily need emotional support (12). Further, the present conceptualization can be used to improve prevention and treatment of maladaptive emotions in patients with cancer. For example, attention-focused interventions could help patients to keep somatic, cancer(treatment)-related symptoms separate from emotional symptoms. Reconsidering life goals or finding alternative ways to achieve life goals may help to prevent emotional symptoms forming a tightly connected network of symptoms. The present conceptualization can also be used to improve adaptive functioning: oncologist and nurses have a critical role in supporting patients in this regard (12, 72). Of course, these potential clinical applications require that empirical support is garnered for the present theory on emotions in patients with cancer, and in turn must themselves be empirically evaluated.

## Author contributions

JD: Writing – original draft. ED: Writing – review & editing. AB: Writing – review & editing. FL: Writing – review & editing. MvL: Writing – review & editing. HV: Writing – review & editing. MS: Writing – review & editing. AB: Writing – review & editing.
